# Physiological and biochemical differences in diapause and non-diapause pupae of *Sericinus montelus* (Lepidoptera: Papilionidae)

**DOI:** 10.3389/fphys.2022.1031654

**Published:** 2022-11-02

**Authors:** Quan-Hong Xiao, Zhe He, Rong-Wei Wu, Dao-Hong Zhu

**Affiliations:** ^1^ Laboratory of Insect Behavior and Evolutionary Ecology, College of Life Science and Technology, Central South University of Forestry and Technology (CSUFT), Changsha, China; ^2^ College of Physical Education, Central South University of Forestry and Technology (CSUFT), Changsha, China

**Keywords:** *Sericinus montelus*, diapause, supercooling point, cryoprotectant, trehalose, water content

## Abstract

The swallowtail butterfly, *Sericinus montelus* Gray, is endemic to East Asia, has high ornamental value but faces an increased risk of extinction. To understand the overwintering strategies of this species, the dynamic changes in supercooling point (SCP) and water and biochemical contents of diapause-destined and non-diapause *S. montelus* pupae were investigated. The SCP of laboratory-reared diapause pupae was as low as −26°C compared to −24°C in diapause pupae in the field. Although there was no significant difference in total water content between diapause-destined and non-diapause pupae, the free water of diapause-destined pupae was significantly lower, and the bound water was significantly higher, than that of non-diapause pupae. Lipid, glycogen, and protein contents of diapause-destined pupae showed a downward trend, whereas the total sugar content showed the opposite trend after pupation. The glycogen content decreased rapidly during the initial stage of pupation, whereas the lipid content decreased significantly after 30 days of pupation, suggesting that diapause-destined pupae deplete glycogen stores during the pre-diapause period and then switch to using lipids during the diapause maintenance phase. Trehalose levels in diapause-destined pupae increased significantly and remained high after pupation. Meanwhile, the trehalose content of overwintering pupae during the diapause maintenance period was significantly higher than that of diapause termination pupae in the field. These results suggest that trehalose is the main cryoprotectant for overwintering pupae. Thus, diapausing *S. montelus* pupae appear to be freeze avoidant, accumulate trehalose as a cryoprotectant, and reduce the free water content to decrease the SCP, enhancing their cold tolerance.

## Introduction

Diapause is widespread in insects and occurs at a specific developmental stage, enabling the animal to avoid adverse environmental conditions (Denlinger 1991; Danks 2007). Generally, diapausing insects have stronger tolerance of adverse environmental conditions, including low temperatures during winter (Holmstrup et al., 2010; Cira et al., 2018). Insects use three main strategies for survival when diapausing at low temperatures: freeze avoidance, freeze tolerance, and chill susceptible (Sinclair et al., 2015; Overgaard and Macmillan, 2017). Freeze-avoidant insects can tolerate moderate to high sub-zero temperatures, but without internal ice formation. These insects improve their cold tolerance by depressing their spontaneous nucleation temperature (supercooling point, SCP) to as low as −20°C, accompanied by a high supercooling ability (Zachariassen and Kristiansen 2000; Bale 2002; Zachariassen et al., 2004). They usually achieve this by accumulating low-molecular-weight carbohydrates and polyols (Xiao et al., 2015; Hasanvand et al., 2020), synthesis of antifreeze proteins (Li, 2012; Duman, 2015), regulation of water content (Zachariassen et al., 2008; Ciancio et al., 2021), and by removing ice nucleators (Tattersall et al., 2012; Costanzo and Lee, 2013).

The swallowtail butterfly, *Sericinus montelus* Gray, is endemic to East Asia, where it is found across China, the Korean Peninsula, Japan, and the Russian Far East, and its host plants are *Aristolochia debilis* Sieb. & Zucc. and *A. contorta* Bunge (Aristolochiaceae) (Chou, 1998). This butterfly has high ornamental value, but faces an increased risk of extinction (Yang et al., 2008; Li et al., 2016). The populations of *S. montelus* have a rather patchy distribution, and their host plants grow along the low banks of fields, irrigation ditches and paths are highly susceptible to human activities (Li et al., 2016). It is a multivoltine insect species, with the number of generations per year varying according to latitude. In China, two generations per year occur in Heilongjiang province (north), compared with six generations in Hubei and Hunan provinces (south) (Luo et al., 2005; Wang et al., 2007; Luo and Zhu, 2009). In Changsha City, Hunan Province, *S. montelus* overwintering pupae emerge as adults in April, and 6th generation larvae pupate in October (Luo and Zhu, 2009). Pupal diapause is induced by the photoperiod during the larval stage (Tani, 1994; Wang et al., 2007; Luo and Zhu, 2009), and shows geographic variation in both the crucial photoperiod for diapause induction and in diapause intensity among different populations of *S. montelus* (Wang et al., 2012). Wang et al. (2007) reported that lipids and carbohydrates were the main metabolic reserves of diapausing pupae in this species.

In the current study, we measured SCP, analyzed the dynamic metabolic process associated with the water content, and levels of sugars, glycogen, lipid, protein, and low-molecular-weight cryoprotectants in diapause-destined and non-diapause pupae of *S. montelus*. These results provide a better understanding of the dynamic pattern of the physical and biochemical metabolic overwintering strategy during winter diapause in this butterfly.

## Materials and methods

### Insect rearing and diapause induction

Adults of *S. montelus* were collected from the suburbs of Changsha, Hunan Province, China (28.2^○^N, 113.0^○^E) in June, 2018. The butterflies were housed in a net chamber (200 cm × 200 cm × 200 cm) in which their host plant, *Aristolochia debilis* Sieb. et Zucc, was growing. *S. montelus* lay their eggs on the back of young leaves or stems of *A. debilis*. The eggs were incubated at 25°C, and newly hatched larvae were reared with fresh *A. debilis* leaves in plastic containers (30 cm × 18 cm × 20 cm) under a light:dark (LD) cycle of 16:8 h and 25°C to establish a laboratory strain.

Pupal diapause of *S. montelus* is induced by the photoperiod during the larval stage (Tani, 1994; Wang et al., 2007; Luo and Zhu, 2009). Luo and Zhu (2009) reported that, when larvae of the Changsha *S. montelus* population were reared at 25°C and LD 12: 12, 73.2% of pupae entered diapause, whereas pupae did not diapause under a LD of 16: 8. The results of preliminary experiments confirmed that all pupae entered diapause at 25°C and LD 10: 14. Thus, in this study, hatching larvae were reared at 25°C and LD 10: 14 or 16: 8 to obtain diapause and non-diapause pupae, respectively.

### Determination of supercooling point

The SCP of *S. montelus* was determined for diapause-destined pupae and non-diapause pupae (*n* = 30 per treatment). SCP was measured by using a thermocouple connected to an automatic temperature recorder (SUN-V, Pengcheng Electronic Technology Co., Ltd., Beijing, China). Individual pupae were placed in a 1.5-ml centrifuge tube filled with degreasing cotton to put the body surface in close contact with the probe. The tube was wrapped in cotton and placed in an ultra-low temperature refrigerator (DW-FL35, Meiling Low Temperature Technology Co., Ltd., Hefei, China) to slowly lower the temperature of the insects. The initiation of freezing was detected as a sudden temperature increase resulting from the release of heat of fusion from body water being transformed into ice; the lowest temperature recorded before the temperature increase was taken as the SCP.

### Determination of water content

The water content of *S. montelus* pupae was determined using the method described by Han et al. (2005), with alterations. A fresh mass (FM) of pupae (*n* = 15 per treatment) was weighed on an electronic balance (Mettler-Toledo Group, 0.0001 g, Zurich, Switzerland), dried at 60°C for 24 h, and then weighed again to determine the dry mass (DM) following the removal of free water. The pupae were then incubated at 60°C to a constant weight (CW) to remove bound water. For diapause pupae, the longer the period after pupation, the longer the drying time to constant weight, taking 36 h 1 day after pupation and 144 h 30 days after pupation.

The different water contents were calculated as follows:

free water content (%)=(FM – DM) / FM×100%

bound water content (%)=(DM – CW) / FM×100%

total water content (%)=(FM – CW) / FM×100%

### Determination of total sugars and glycogen

The total sugars and glycogen were extracted according to the method of Zhou et al. (2004). Individual pupae were weighed and then boiled in a water bath in a 10 ml tube for 1 h. After cooling, 100 μl saturated Na_2_SO_4_ and 200 μl methanol were added to the tube and the pupa was then mashed with a glass rod, which was then rinsed with 100 μl distilled water and 3 ml chloroform-methanol (methanol: chloroform = 1: 1). After stratification, 300 μL methanol was added and mixed evenly. Samples were then centrifuged at 2,500 r/min for 5 min, and the supernatant was removed. After adding 1 ml of 66% ethanol containing saturated Na_2_SO_4_ to the pellet, the samples were centrifuged at 12,000 × *g* for 5 min, and the pellet was then re-extracted with 0.5 ml of 66% ethanol containing saturated Na_2_SO_4_; the supernatants were combined to determine the total sugar content.

The extracted precipitation was placed in a 55°C water bath for 5 min; 0.5 ml of 30% KOH was then added and the water bath was set to boil for 20 min. Next, 1 ml 95% ethanol was added to each sample, which were then centrifuged at 3,000 r/min for 15 min; the pellet was re-extracted with 0.5 ml distilled water and 1 ml 95% ethanol, and the supernatant was removed after centrifugation. Then, 2 ml distilled water was added to the pellet and boiled in a water bath for glycogen determination.

Total sugars and glycogen were determined by using the anthrone method. Absorbance was measured at 620 nm on a spectrophotometer (G10S UV-Vis, Thermo Fisher Scientific, Shanghai, China) using glucose (Sangon Biotech) as a standard. Twenty individual samples were tested for each treatment group, and the average contents of total sugars and glycogen per milligram of fresh tissue were obtained.

### Lipid content

The lipid content of *S. montelus* pupae was measured according to the method described by Colinet et al. (2006). The pupae were dried to a constant weight at 60°C, and their dry mass (DM_1_) was determined (*n* = 15 per treatment). Individual pupae were thoroughly ground, and 2 ml of chloroform-methanol (chloroform: methanol = 2: 1) was added. The homogenate was left for 24 h, and then centrifuged at 5,000 r/min for 10 min. After transferring the supernatant, the remaining precipitation was dried to a constant weight (DM_2_) in a 60°C incubator. The total lipid content was calculated as:

$$Total\;lipid\;content\;\left(\%\right)=\left({DM}_{1}\;–\;{DM}_{2}\right)\;/\;{DM}_{1}\times 100\%$$

### Protein content

Individual pupae were weighed and then precooled normal saline was added at a weight (g): volume (ml) ratio of 1: 10, followed by homogenization in a high-speed grinder. The samples were centrifuged at 5,000 r/min for 10 min, and the supernatant was used to test the protein content. Protein determination was performed by Coomassie Blue staining method, using a total protein determination kit (Jiancheng Bioengineering Institute, Nanjing, China) according to the manufacturer’s instructions. Absorption was measured at 595 nm. Twenty individual samples were tested for each treatment group.

### Low-molecular-weight cryoprotectants

The levels of trehalose, glucose, sorbitol, sucrose, glycerol, mannitol, inositol, and ribitol were determined in diapause-destined and non-diapause pupae (*n* = 20 per treatment). Individual pupae were homogenized with 80% ethanol and centrifuged at 12,000 × *g* for 15 min at 4°C. Then, the supernatant was dried at 35°C in a vacuum drying oven. Each sample was resuspended in 0.15–0.2 ml redistilled water and cleaned by passing them through a 0.22 μm syringe filter. Analyses of cryoprotectants were performed by high-performance liquid chromatography (Ultimate 3,000, Thermo Fisher Scientific, Shanghai, China) with an RI detector using a Ca column (30 cm × 7.8 mm, SUPELCOGEL Ca, Merck, United States). The mobile phase was redistilled water, and separation was achieved at 75°C. The injection volume was 20 μl and the flow rate was 0.5 ml/min.

### Determination of seasonal changes in supercooling point and cryoprotectants

Newly hatched larvae of *S. montelus* were reared with *A. debilis* and placed in the nursery garden at CSUFT under natural conditions. To find out the seasonal changes of SCP and cryoprotectants in field, pupae were sampled randomly from the nursery garden on the 15th of November, January, and March, and then their SCP and cryoprotectants were detected using the above method.

### Data analysis

Statistical software SPSS 25.0 (IBM Corp.) was used for statistical analysis of the obtained data, which were expressed as means ± SE. Differences of SCP and the contents of water, energy substances and cryoprotectants between diapause-destined and non-diapause pupae groups were evaluated using Student’s *t*-tests. The one-way analysis of variance (ANOVA) and the post hoc Tukey’s test were subsequently run to compare multiple treatments of diapause-destined, non-diapause and field pupae groups, respectively. *p* < 0.05 was considered statistically significant.

## Results

### Supercooling point

**FIGURE 1:**
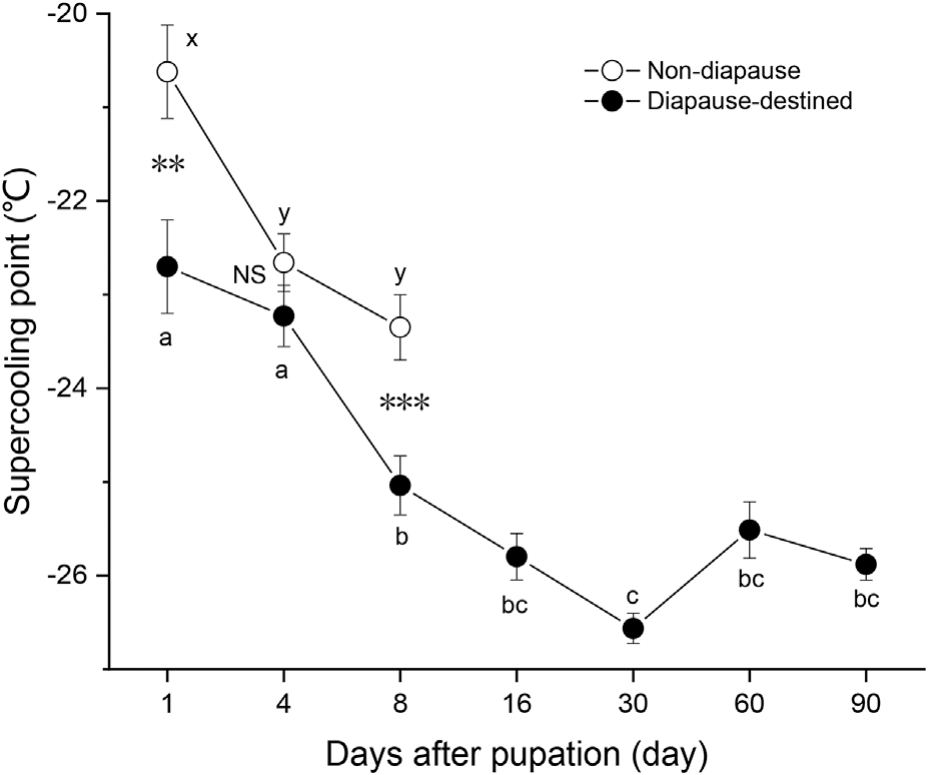
Changes in the supercooling point of diapause-destined and non-diapause *Sericinus montelus* pupae after pupation. Each point shows the mean ± SE, *n* = 30 each. Error bars with the same letters indicate no significant difference at *p* = 0.05 using one-way ANOVA followed by Tukey’s multiple comparison test. NS, not significant (*p* > 0.05), **p < 0.01, ***p < 0.001 by Student’s *t*-tests between diapause-destined and non-diapause.

To obtain diapause pupae, newly hatched larvae of *S. montelus* were reared under short daylength conditions (LD 10: 14 h) at 25°C. SCPs of diapause-destined pupae and non-diapause pupae were measured (Figure 1). Non-diapause pupae began emerging on day 9 after pupation, and their SCPs were examined until day 8 after pupation. Diapause-destined pupae was examined until 90 days after pupation to clarify any dynamic changes in their SCP. The SCPs of diapause-destined pupae gradually decreased (*F*_6, 203_ = 22.019, *p* < 0.0001) to the lowest level (–26.6 ± 0.16°C, means ± SE) at day 30 after pupation. From 8 to 90 days after pupation, SCP stabilized at around –26°C, indicating that the pupae were in the diapause maintenance phase. On day 8 after pupation, the SCP of diapause pupae was significantly lower than that of non-diapause pupae (Student’s *t*-test, *p* = 0.001).

### Body water content

**FIGURE 2:**
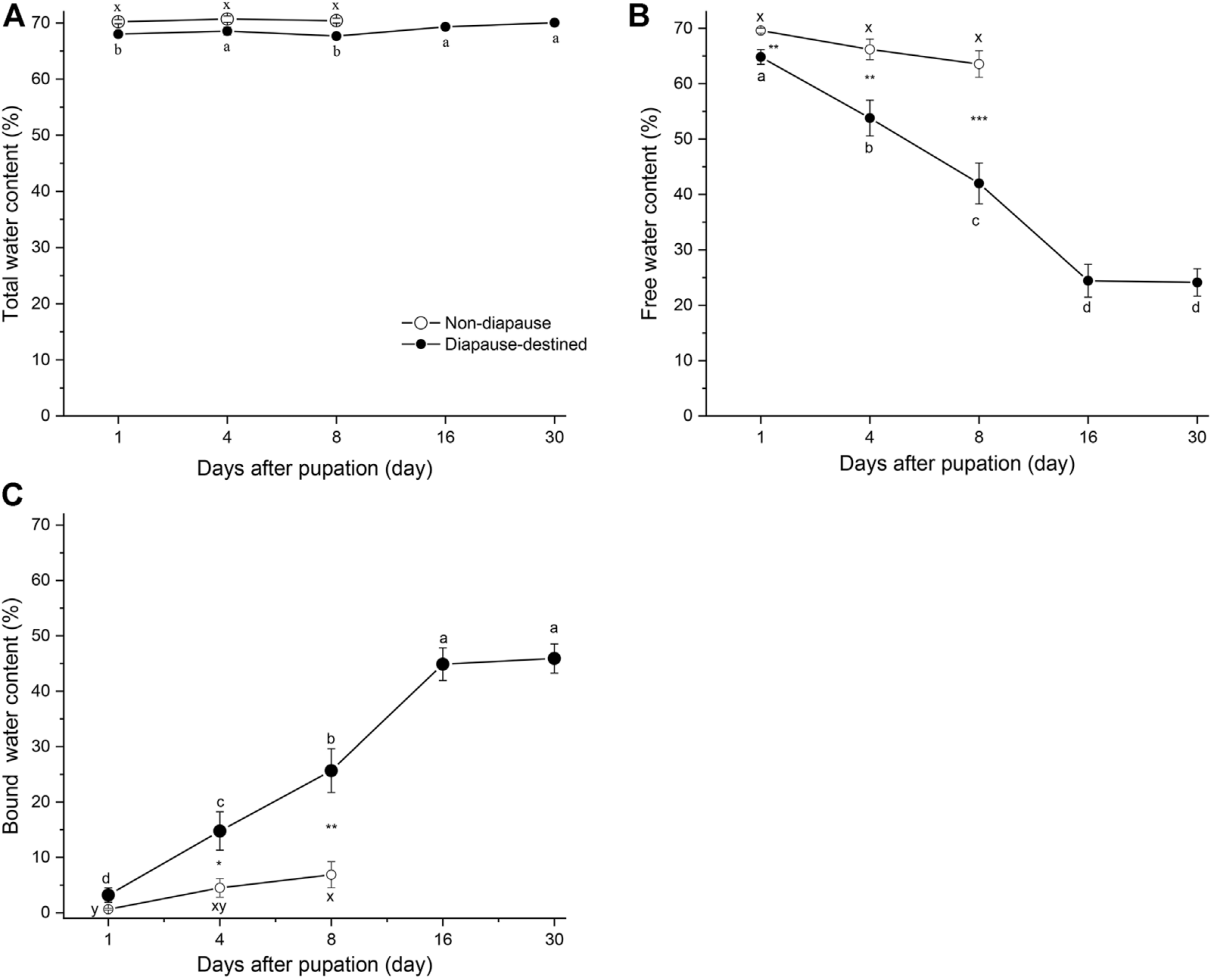
Changes in total water **(A)**, free water **(B)**, and bound water content **(C)** of diapause-destined and non-diapause *Sericinus montelus* pupae after pupation. Each point shows the mean ± SE, *n* = 15 each. Error bars with the same letters indicate no significant difference at *p* = 0.05 using one-way ANOVA followed by Tukey’s multiple comparison test. *p < 0.05, **p < 0.01, ***p < 0.001 by Student’s *t*-tests between diapause-destined and non-diapause.

To determine the contents of free and bound water in diapause-destined and non-diapause pupae, the pupae of *S. montelus* were incubated at 60°C for 24 h or to a constant weight. No significant difference in free water content (*F*_2, 42_ = 2.910, *p* = 0.066) and only slight changes in the bound water content (*F*_2, 42_ = 3.533, *p* = 0.038) of non-diapause pupae were observed before adult emergence (Figures 2B, C). By contrast, the free water (*F*_4, 42_ = 39.620, *p* < 0.0001) and bound water (*F*_4, 42_ = 38.738, *p* < 0.0001) of diapause-destined pupae varied significantly after pupation: the free water content gradually decreased until stabilizing at ∼25%, whereas the bound water content gradually increased until stabilizing at ∼45% on day 16 after pupation. The free water content of diapause-destined pupae was significantly lower than that of non-diapause pupae (Student’s *t*-test, *p* < 0.01), and the bound water content of diapause-destined pupae was significantly higher than that of non-diapause pupae except on day 1 after pupation (Student’s *t*-test, *p* > 0.05 for day 1, remaining: *p* < 0.05 or < 0.01) (Figures 2B, C).

The total water content of non-diapause pupae did not change significantly (*F*_4, 42_ = 0.274, *p* = 0.762), and diapause-destined pupae slightly varied from 1 to 30 days after pupation (*F*_4, 42_ = 3.64, *p* < 0.01). There were no significant differences between diapause and non-diapause pupae at days 1, 4, and 8 after pupation (Student’s *t*-test, *p* > 0.05) (Figure 2A).

### Energy substances

**FIGURE 3:**
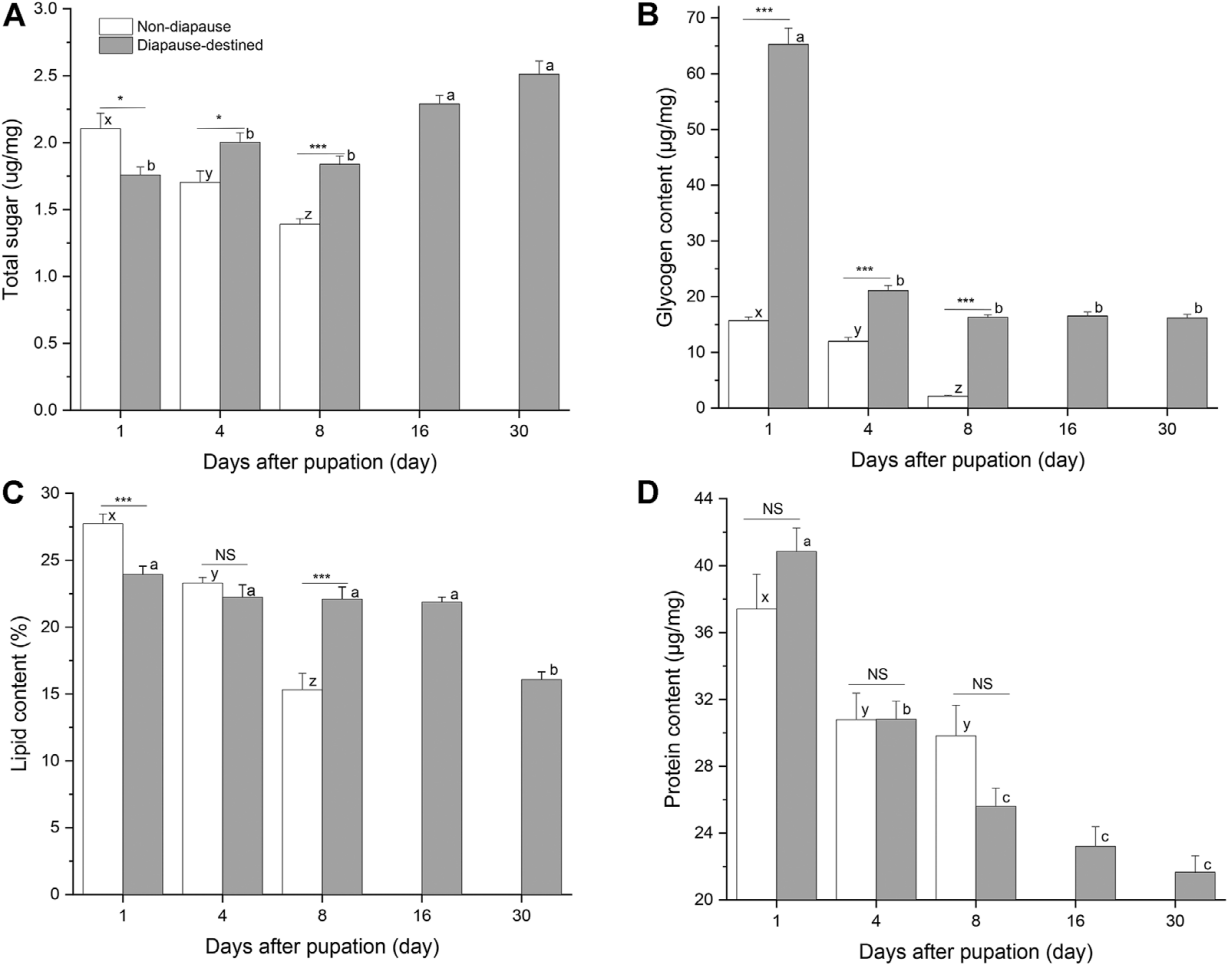
Total sugars **(A)**, glycogen **(B)**, Lipid **(C)** and protein contents **(D)** in diapause-destined and non-diapause *Sericinus montelus* pupae. Error bars indicate SE, *n* = 15–20 each. Bars with the same letters indicate no significant difference at *p* = 0.05 using one-way ANOVA followed by Tukey’s multiple comparison test. NS, no significant (*p* > 0.05), *p < 0.05, ***p < 0.001 by Student’s *t*-tests between diapause-destined and non-diapause.

Energy stores were measured by quantifying soluble protein, neutral lipids, total sugars, and glycogen levels in individual diapause-destined and non-diapause *S. montelus* (Figure 3). The total sugar (*F*_2, 57_ = 17.330, *p* < 0.0001), lipid (*F*_2, 42_ = 53.162, *p* < 0.0001), and glycogen contents (*F*_2, 57_ = 151.369, *p* < 0.0001) of non-diapause pupae decreased rapidly before adult emergence. For diapause-destined pupae, the total sugar content showed an upward trend after pupation, and was significantly higher after day 16 than during the early pupal stage (*F*_4, 95_ = 18.813, *p* < 0.0001). Furthermore, the concentration of the total sugar content of diapause-destined pupae was significantly higher than that of non-diapause pupae at day 4 and 8 after pupation (Student’s *t*-test, *p* < 0.05 for day 4, *p* < 0.001 for day 8) (Figure 3A). The changes in the glycogen and lipid contents of diapause-destined pupae were inverse to those seen for the total sugar content. At the initial stage of pupation (day 1), the glycogen content peaked at 65.28 ± 2.92 μg/mg (mean ± SE), four times higher than that of the non-diapause pupae. However, it rapidly decreased to ∼16 μg/mg and remained stable during the diapause maintenance period. This was still significantly higher than that of non-diapause pupae on day 4 and 8 after pupation (*t*-test, each *p* < 0.001) (Figure 3B). Lipid contents of diapause-destined pupae showed a significant change (*F*_4, 70_ = 18.005*, p <* 0.0001), with decreases occurring 30 days after pupation. On day 8 after pupation, the lipid content of diapause pupae was significantly higher than that of non-diapause pupae (Student’s *t*-test, each *p* < 0.001) (Figure 3C).

The protein content of both diapause-destined (*F*_4, 95_ = 44.938, *p* < 0.0001) and non-diapause pupae (*F*_2, 57_ = 5.038, *p* = 0.01) decreased after pupation, although the amount in non-diapause pupae remained at ∼30 μg/mg after day 4, suggesting that they entered reproductive development. There were no significant differences between diapause-destined and non-diapause pupae at days 1, 4, and 8 after pupation (Student’s *t*-test, *p* > 0.05) (Figure 3D).

### Low-molecular-weight cryoprotectants

**FIGURE 4:**
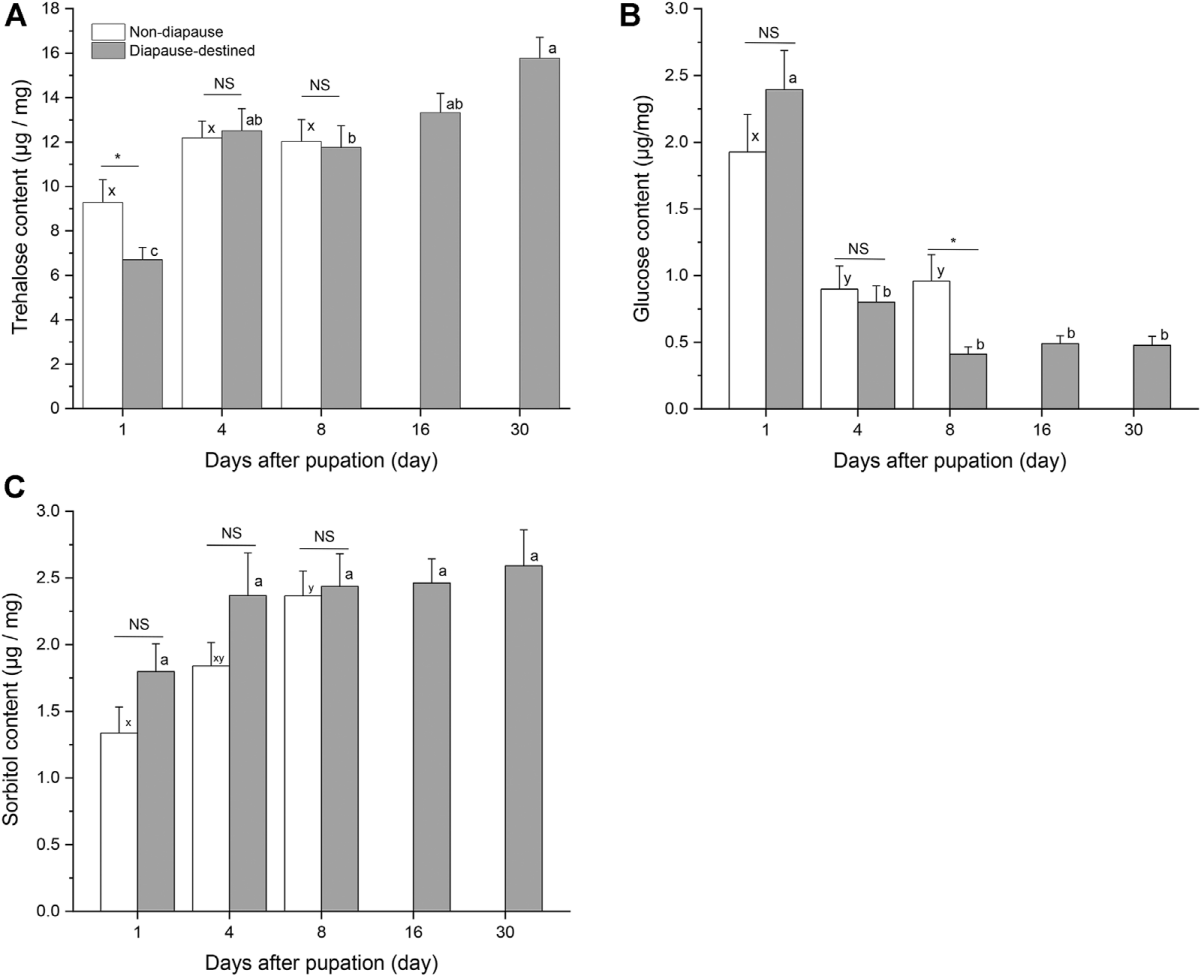
Trehalose **(A)**, glucose **(B)** and sorbitol contents **(C)** in diapause-destined and non-diapause *Sericinus* pupae. Error bars indicate SE, *n* = 20 each. Bars with the same letters indicate no significant difference at *p* = 0.05 using one-way ANOVA followed by Tukey’s multiple comparison test. NS, no significant (*p* > 0.05), *p < 0.05 by Student’s *t*-tests between diapause-destined and non-diapause.

Attempts were made to detect eight potential cryoprotectants in *S. montelus* pupae; however, it was not possible to obtain data for five of these (sucrose, glycerol, mannitol, inositol, and ribitol) because of their low concentrations. At the start of pupation, there was a high level of glucose in diapause-destined pupae, which then decreased rapidly from day 4 (*F*_4, 95_ = 31.646, *p* < 0.0001), becoming lower even than that of non-diapause pupae by day 8 after pupation (Student’s *t*-test, *p* < 0.05) (Figure 4B). Sorbitol content of diapause-destined pupae remained stable after pupation (*F*_4, 95_ = 1.477, *p* > 0.05), and there was no significant difference between diapause pupae and non-diapause pupae (Student’s *t*-test, *p* > 0.05) (Figure 4C). Neither was there a significant difference in trehalose content between diapause-destined and non-diapause pupae at days 4 and 8 after pupation (Student’s *t*-test, *p* > 0.05). However, the trehalose content of diapause-destined pupae increased from 6.70 ± 0.55 μg/mg at the start of pupation to 15.78 ± 0.94 μg/mg at day 30 after pupation, a significant change (*F*_4, 95_ = 14.276, *p* < 0.0001) (Figure 4A).

### Seasonal changes in supercooling points and cryoprotectants

**FIGURE 5:**
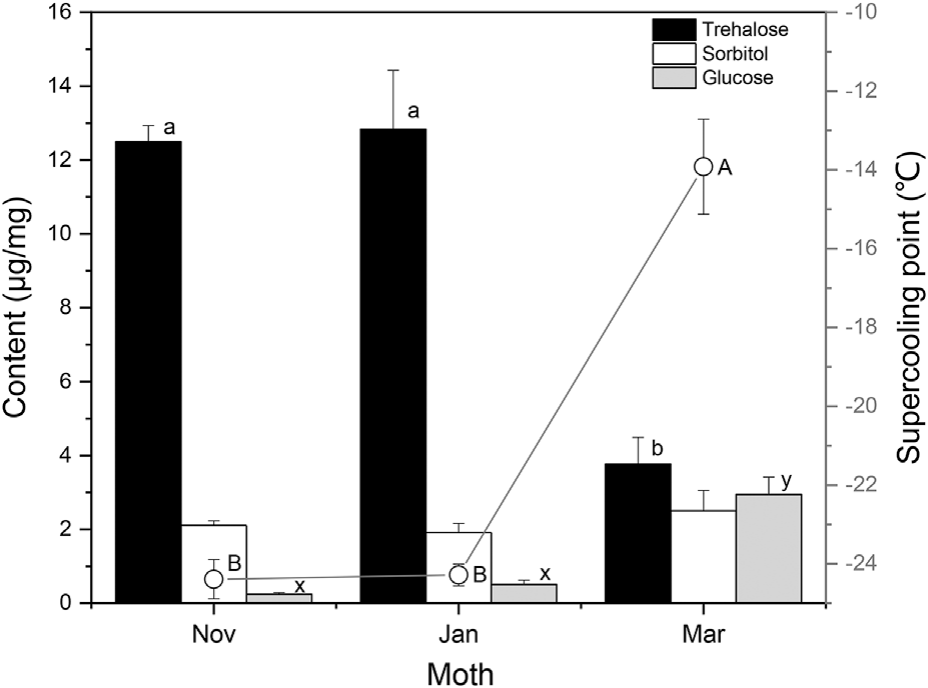
Seasonal changes in the contents of low-molecular-weight cryoprotectants and supercooling points (curve) of overwintering *Sericinus montelus* pupae in the field. Error bars indicate SE, *n* = 15 each. Bars with the same letters indicate no significant difference at *p* = 0.05 using one-way ANOVA followed by Tukey’s multiple comparison test.

The Changsha population of *S. montelus* has six generations in a year, with the overwintering generation pupating during mid–late October, and adults emerging in mid-April (Luo and Zhu, 2009). For comparison with the results of laboratory-reared insects, the variation in SCP and cryoprotectants in field overwintering pupae of *S. montelus* was determined (Figure 5). Sorbitol and glucose remained at low levels during diapause maintenance in winter, and glucose was significantly elevated (*F*_2, 42_ = 28.113, *p* < 0.0001), whereas sorbitol showed no change (*F*_2, 35_ = 0.796, *p* > 0.05) by the following spring. Trehalose remained at a high level from November to January during the diapause maintenance period, but decreased significantly from 12.50 ± 0.44 μg/mg in November to 3.76 ± 0.72 μg/mg in March (*F*_2, 42_ = 24.206, *p* < 0.0001). Correspondingly, SCPs of diapause maintenance pupae remained stable in November to January, but increased significantly from –24.4 ± 0.50°C in November to –13.9 ± 1.2°C in March (*F*_2, 87_ = 60.817, *p* < 0.0001).

## Discussion

Overwintering insects are exposed to prolonged stress conditions, particularly low temperatures, starvation, and desiccation. They usually undergo complex adaptive changes involving physiological, biochemical, and endocrine regulation (Denlinger, 2002). In the current study, the dynamic changes in SCP and the water and biochemical content of diapause-destined and non-diapause pupae of *S. montelus* were compared. The cold hardiness of insects can be measured by their SCP and cold tolerance strategies are categorized as freeze avoidant, freeze tolerance, and chill susceptible (Sinclair et al., 2015; Overgaard and Macmillan, 2017). The minimum SCP of laboratory-reared diapause *S. montelus* pupae was –26°C, and that of diapause pupae in the field was –24°C (Figures 1, 5). Changsha is located in southern China. Its winter climate is relatively mild, and the minimum temperature is generally not less than –5°C. Laboratory-obtained diapause pupae of *S. montelus* treated with –24°C for 2 h resulted in an 20% survival rate (unpublished data). Therefore, the cold tolerance strategy of *S. montelus* is likely to be freeze avoidant. Similar conclusions were reached in previous studies on other butterfly species, including *Papilio machaon* (Hirai et al., 2016) and *Papilio zelicaon* (Williams et al., 2014).

Both diapausing and non-diapausing insects store metabolic reserves of the same three macronutrient groups: lipids, carbohydrates, and proteins. However, compared with non-diapause individuals, the quantity and quality of nutrient stores in diapause-destined insects often change noticeably, with diapause individuals entering an alternative developmental pathway with its own metabolic demands (Kostál, 2006; Hahn and Denlinger, 2011). The current results showed that the total sugars, glycogen, and lipids of non-diapause *S. montelus* pupae were rapidly consumed, whereas the protein content tended to stabilize after the fourth day of pupation, indicating that they entered the developmental pathway of tissue remodeling and reproductive preparation. By contrast, in diapause-destined pupae, the lipid, glycogen, and protein contents decreased, whereas the total sugar content increased after pupation. The glycogen content of diapause-destined pupae was four times as high as that of non-diapause pupae at the initial pupation stage, and then rapidly decreased (Figure 3). In addition to longer-term shifts in nutrient use, rapid changes also occur (Hahn and Denlinger, 2011). The rapidly decreasing glycogen might be converted into simple sugars or sugar alcohol, as reported by several previous studies in Lepidoptera [e.g., *Ocneria terebinthina* (Behroozi et al., 2012), *Ectomyelois ceratoniae* (Heydari and Izadi, 2014), and *Kermania pistaciella* (Mollaei et al., 2016)]. The lipid content remained stable during the early stage of diapause maintenance and decreased significantly after 30 days of pupation. These results suggest that diapause-destined *S. montelus* pupae deplete glycogen stores during the pre-diapause period and then switch to use lipids during the diapause maintenance phase. Adult diapause of the mosquito *Culex pipiens* shows a similar pattern (Zhou and Miesfeld, 2009). Previous research indicated that the lipid and carbohydrate contents of diapause *S. montelus* pupae were significantly higher than those of non-diapause pupae, but the protein content was not significantly different (Wang et al., 2007). Lipids, carbohydrates, and proteins are major forms of energy reserves in diapause insects (Hahn and Denlinger 2007). The results of the current study showed that the lipid, glycogen, and total sugar contents of diapause pupae during the diapause maintenance phase (8 days after pupation) were significantly higher than those of non-diapause pupae, indicating that diapausing pupae have the ability to reserve energy in the form of lipids, glycogen, and sugars, and use it during periods of repressed metabolism while they are overwintering.

Many overwintering insects improve their cold hardiness by synthesizing and accumulating low-molecular-weight cryoprotectants (Zachariassen et al., 2008; Xiao et al., 2015; Izadi et al., 2019; Hasanvand et al., 2020; Ciancio et al., 2021). Several studies have confirmed that increased cold hardiness is mainly associated with increased trehalose and/or glycerol, sorbitol, and glucose in lepidopterans (Pullin and Bale, 1989; Kukal et al., 1991; Williams et al., 2014; Zheng et al., 2014; Xiao et al., 2015; Vrba et al., 2017). We analyzed the concentrations of eight low-molecular-weight sugars and polyols in *S. montelus* pupae, and found that trehalose in diapause-destined pupae increased significantly and remained at a high level after pupation. The trehalose content of overwintering pupae during the diapause maintenance period was significantly higher than that of diapause termination pupae in the field (Figures 4, 5). These results suggest that trehalose is the main cryoprotectant for overwintering *S. montelus* pupae. Interestingly, the increasing trend in trehalose content in diapause-destined pupae was consistent with the decreasing trend in SCP. Especially in field conditions, trehalose levels remained high from November to January and decreased significantly in March, whereas SCP changed in the opposite direction. This suggests that diapausing *S. montelus* pupae accumulate trehalose as a cryoprotectant to decrease their SCP and enhance their cold tolerance.

Cryoprotective dehydration is one strategy to improve cold tolerance in some insects (Kawarasaki et al., 2013; Ciancio et al., 2021). Several studies have shown that the water content of non-diapause insects is significantly higher than that of diapause individuals, such as *Pieris melete* (Xiao et al., 2015), *Ectomyelois ceratoniae* (Heydari and Izadi, 2014), and *Streltzoviella insularis* (Pei et al., 2020). In *S. montelus*, Wang et al. (2007) reached similar conclusions*.* By contrast, some overwintering insects exhibit a similar water content between diapausing and non-diapausing individuals, such as *Mayetiola destructor* (Benoit et al., 2010) and *Eurygaster integriceps* (Hasanvand et al., 2020). The results of the current study showed that the free water of diapause-destined *S. montelus* pupae decreased obviously after pupation, and was significantly lower than that of non-diapause pupae, and *vice versa* for the bound water content. However, the total water content of diapause-destined pupae showed little change and was not significantly different to that in non-diapause pupae (Figure 2). Winter in temperate environments is dry and overwintering pupae do not supplement water by eating or drinking for several months. Our unpublished observations showed that, with the increase in diapause pupal age, the pupal integument of *S. montelus* becomes denser, tougher, and less likely to break, which could be related to the prevention of excessive water loss. Therefore, *S. montelus* diapause pupae might decrease their free water content to improve their cold tolerance and increase their bound water content to maintain a high total water content to ensure that they contain enough water for subsequent metabolism and development.

In conclusion, our study shows a dynamic profile of SCP, content of water, and energy substance reserves, as well as accumulating low-molecular-weight cryoprotectants in diapausing *S. montelus* pupae. The butterfly reserved energy in the form of lipids, glycogen and sugars, and utilizes these to compensate for a repressed metabolism during the overwintering period. Diapausing pupae of *S. montelus* were freeze avoidant, accumulated trehalose as a cryoprotectant, and reduced their free water content to decrease their SCP, enhancing their cold tolerance.

## Data Availability

The original contributions presented in the study are included in the article/supplementary material, further inquiries can be directed to the corresponding author.
